# A Comprehensive Summary of the Current Understanding of the Relationship between Severe Obesity, Metabolic Syndrome, and Inflammatory Status

**DOI:** 10.3390/jcm12113818

**Published:** 2023-06-02

**Authors:** Razvan-Marius Ion, Melania Sibianu, Adina Hutanu, Felicia Gabriela Beresescu, Daniela Tatiana Sala, Mocian Flavius, Ancuta Rosca, Calin Constantin, Alexandra Scurtu, Renata Moriczi, Mircea Gabriel Muresan, Popescu Gabriel, Raluca Niculescu, Radu Mircea Neagoe

**Affiliations:** 1 Doctoral School of Medicine and Pharmacy, George Emil Palade University of Medicine, Pharmacy, Science, and Technology of Targu Mures, 540142 Targu Mures, Romania; 2 Clinical Medicine I, Mures County Emergency Hospital, 540136 Targu Mures, Romania; 3 Department of Laboratory Medicine, George Emil Palade University of Medicine, Pharmacy, Science, and Technology of Targu Mures, 540142 Targu Mures, Romania; 4 Department of Morphology of Teeth and Dental Arches, Faculty of Dentistry, George Emil Palade University of Medicine, Pharmacy, Science, and Technology of Targu Mures, 540142 Targu Mures, Romania; 5 Second Department of Surgery, George Emil Palade University of Medicine, Pharmacy, Science, and Technology of Targu Mures, 540139 Targu Mures, Romania; 6 Third Department of Surgery, George Emil Palade University of Medicine, Pharmacy, Science, and Technology of Targu Mures, 540139 Targu Mures, Romania; 7 Second Department of Surgery, Mures County Emergency Hospital, 540136 Targu Mures, Romania

**Keywords:** morbid obesity, pro-inflammatory status, metabolic syndrome, weight-loss effects

## Abstract

At present, obesity, as a part of metabolic syndrome, represents the leading factor for disability, and is correlated with higher inflammation status, morbidity, and mortality. The purpose of our study is to add new insights to the present body of knowledge regarding the correlations between chronic systemic inflammation and severe obesity, which cannot be treated without considering other metabolic syndrome conditions. Biomarkers of high-level chronic inflammation are recognized as important predictors of pro-inflammatory disease. Besides the well-known pro-inflammatory cytokines, such as WBCs (white blood cells), IL-1 (interleukin-1), IL-6 (interleukin-6), TNF-alpha (tumor necrosis factor-alpha), and hsCRP (high-sensitivity C-reactive protein), as well as anti-inflammatory markers, such as adiponectin and systemic inflammation, can be determined by a variety of blood tests as a largely available and inexpensive inflammatory biomarker tool. A few parameters, such as the neutrophil-to-lymphocyte ratio; the level of cholesterol 25-hydroxylase, which is part of the macrophage-enriched metabolic network in adipose tissue; or levels of glutamine, an immune–metabolic regulator in white adipose tissue, are markers that link obesity to inflammation. Through this narrative review, we try to emphasize the influence of the weight-loss process in reducing obesity-related pro-inflammatory status and associated comorbidities. All data from the presented studies report positive results following weight-loss procedures while improving overall health, an effect that lasts over time, as far as the existing research data show.

## 1. Introduction

According to the World Health Organization (WHO) European Regional Obesity Report in 2022, overweight and obesity have reached epidemic proportions in Europe, affecting 58% of adults and about one in three children, and the report also shows that the disease causes 200,000 cases of cancer and 1.2 million deaths per year [1]. At present, obesity is the leading factor for disability, and it has been correlated with higher morbidity and mortality since the onset of the COVID-19 pandemic [1,2].

The growing prevalence of worldwide obesity is still a major global health challenge despite all of the warning signs raised in the last two decades leading to the “most prevalent cause of human morbidity and mortality”. Obesity is a condition related to genetic, environmental, and behavioral factors, but even so, it remains a preventable and, more importantly, treatable disease. The danger of this condition comes from the fact that it leads to more serious diseases. On one hand, it is directly related to cardiovascular disease and type 2 diabetes mellitus, and on the other hand, it is involved in the appearance of certain types of cancer, but it affects almost all systems and organs of the body to varying degrees. Furthermore, musculoskeletal complications arise due to the mechanical effects of increased body weight. Excess body fat leads to premature death and is a serious risk factor for disabilities [1,3].

According to a report presented at the 2022 European Congress on Obesity, the incidence of obesity in Europe is the second highest in the world after the Americas [1]. The causes of obesity seem to be more complex than an unhealthy diet and a lack of physical activity. Mental, emotional, and physical stressors; life-specific environmental factors in modern, highly digitalized European societies, such as unhealthy food marketing online; and the preference for a sedentary lifestyle also play an important role [1,4].

The negative effects of excess fat have repercussions on all systems and organs of the body. Weight loss improves inflammation locally and systemically, given that adiposity redundancy is a form of destabilizing aggression against the immune system. A healthy and balanced lifestyle is the key to returning the body to health and equilibrium. 

The purpose of our narrative review is to add new insights to the present body of knowledge regarding the correlations between chronic systemic inflammation and both obesity (i.e., severe obesity) and metabolic syndrome. 

## 2. Materials and Methods

### 2.1. Search Strategy

Our study was conducted by four researchers (I.R.M., M.S., A.H., and R.N.M.) in November–December 2022. A search of the PubMed, Cochrane Library, and ScienceDirect English-language electronic databases was performed using the following search terms: “severe obesity” AND/OR “metabolic syndrome” AND “severe obesity” AND/OR “chronic systemic inflammation” AND “metabolic syndrome” AND/OR “chronic systemic inflammation”, as well as combinations of these terms. Our research included papers published up to 2022, with a special focus on recent publications (January 2018 up to December 2022), mainly prospective research articles, reviews, meta-analyses, comparative papers with a control group, and randomized controlled trials (RCTs); papers before 2018 were included only if they provided important data such as definitions, guidelines, and trustworthy new findings.

The inclusion criteria were as follows: (1) articles containing relevant data regarding the topic; (2) articles containing comparative data; and (3) articles written in English that contain information related to obesity, metabolic syndrome, and systemic chronic inflammation. 

The exclusion criteria were as follows: articles with an insufficient amount of data to analyze; a lack of primary outcomes; a lack of comparative data; and non-English-language studies. We also excluded summary congress reports and abstracts with incomplete data.

**Figure 1:**
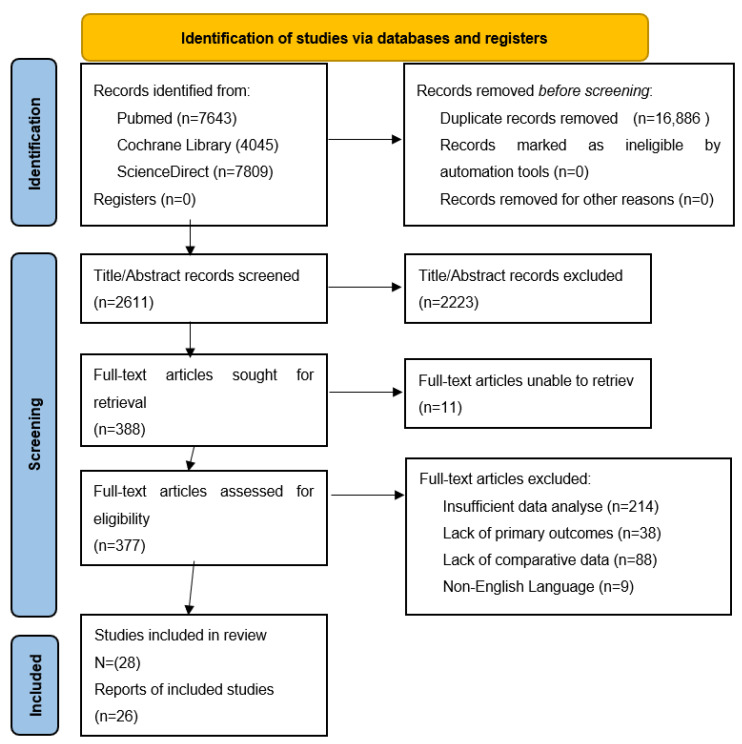
PRISMA 2020 flow diagram for new systematic reviews which included searches of databases and registers only [5].

All data were assessed and included independently by two researchers; the study was performed according to the Preferred Reporting Items for Systematic Reviews (PRISMA) 2020 guidelines [5]. All the articles included in the narrative review were critically appraised using a 10-question CASP checklist. The initial search yielded 19,497 papers; after removing duplicates, abstracts, and non-English studies, we focused our attention on 28 studies. The PRISMA flowchart is presented in Figure 1.

### 2.2. Outcome Measures

The primary endpoint of this review was to analyze correlations between chronic systemic inflammation and both severe obesity and metabolic syndrome. Secondary outcome measures included the role of the weight-loss process, especially after bariatric procedures, in reducing the obesity-related pro-inflammatory status and comorbidities.

## 3. Metabolic Syndrome Exacerbates Comorbidities

The definition of metabolic syndrome (MetS) includes five criteria: (1) central obesity, defined as a large waist circumference (WC), an elevated waist–hip ratio (>0.90 for females and >0.85 for males), or a body mass index (BMI) above 30 kg/m; (2) raised blood pressure; (3) dyslipidemia, i.e., elevation of total cholesterol (TC), low-density lipoprotein (LDL), and/or triglycerides (TG), and low high-density lipoprotein cholesterol (HDL-C); (4) raised fasting plasma glucose; and (5) raised fasting serum insulin. The prevalence of MetS can increase with the prevalence of obesity as the two entities are closely related [6,7].

As previously discussed, obesity predisposes an individual to, in addition to cardiovascular disease (CD) and type 2 diabetes (T2D), a number of serious diseases, such as cholesterol gallstones, obstructive sleep apnea (OSA), non-alcoholic fatty liver disease (NAFLD), non-alcoholic steatohepatitis (NASH), polycystic ovarian syndrome (PCOS), fertility disorders, gout, osteoarthritis, psoriasis, asthma, depression, and dementia. Previous studies show that various cancers, including esophageal, breast, colorectum, liver, pancreas, kidney, gallbladder, prostate/ovary, and endometrial cancer, as well as malignant melanoma, leukemia, multiple myeloma, and meningioma, seem to also be related to obesity [8]. 

Any three of the five abnormal findings fulfill the diagnosis of MetS. As obesity affects atherogenesis due to clinical complications, including ischemic heart disease, peripheral artery disease, cerebrovascular disease, and diabetes mellitus, some indicators for the evaluation of cardiometabolic risk have also been useful [9,10]. 

The accumulation of fat around the abdomen is defined as central obesity and is measured clinically with a measuring tape following the measurement of the circumference of the waist. A healthy female could have up to an 80 cm waist circumference, while a healthy man could have up to a 94 cm waist circumference. A female whose waist measures over 88 cm and a male whose waist measures over 110 cm would be diagnosed with central obesity and may present major health risks [8]. The most accepted classification of excess adiposity is the one made by the WHO, expressed as BMI, which is defined in kg (weight in kilograms)/m^2^ (height in square meters) [1]:

- Grade 1 obesity (commonly referred to as overweight): a BMI between 25 and 29.9 kg/m^2^;
- Grade 2 obesity (called obesity): a BMI between 30 and 39.9 kg/m^2^;
- Grade 3 obesity (referred to as morbid obesity): a BMI above or equal to 40 kg/m^2^ [1,9].

Large-scale epidemiological studies have shown that cardiovascular, metabolic, and cancer morbidity begin to increase from a BMI ≥ 25. A BMI between 25 and 30 should be considered important from a medical point of view and in terms of the need for medical care, especially in the presence of risk factors for adiposity, such as high blood pressure and glucose intolerance [1]. An increased BMI is a marker for MetS and is also used as a screening method to assess cardiometabolic risk factors (hypertension, diabetes, and cardiovascular disease), as well as a predictor of total body fat percentage and visceral fat mass [10].

## 4. Inflammation in Obesity: The Link Mechanism and the Complications

Increased deposits of visceral rather than subcutaneous fat, or central adiposity, is the “particular weight gain model”, while abdominal fat in particular is associated with chronic, low-grade inflammation and immune activation [11]. Visceral fat, known as “deep fat”, has a greater impact on health as it is more biologically active, has a higher density of cells, has greater blood flow, and is located closer to the portal vein, which results in an increased level of fatty acids reaching the liver [1,9]. 

Mammalians have three distinctive types of fat tissue: white adipose tissue (WAT), brown adipose tissue (BAT), and beige adipose tissue, with totally different biological functions. While WAT is specialized in energy storage and mobilization, hormone secretion, and immune function, BAT is specialized in energy consumption and utilizes chemical energy, which plays an essential part in the maintenance of central body temperature, i.e., thermogenesis [12,13].

An occurrence known as the “browning of WAT” was discovered through the activation or cold exposure of β-adrenergic receptors (β-ARs) shaping the so-called beige adipose tissue. By absorbing sugars and fatty acids to produce caloric heat, both brown and beige adipocytes play a major role in regulating glucose and lipid metabolism [14]. Cheng et al. confirmed the fact that the stimulation of BAT and beige AT may represent a possible strategy to treat excessive adiposity and diabetes. Research confirms that active BAT plays a major role in improving glucose and lipid metabolism. It improves glucose tolerance, insulin sensitivity, and pancreatic beta-cell function and reduces the need for insulin secretion [15].

Firstly, white fat cells widely distributed throughout the body store excess energy as triglycerides [11]. When the body needs this stored energy elsewhere, it is released as free fatty acids. Secondly, fat cells form an active metabolic organ. Adipocytes generate an impact on pancreatic beta-cell function, hepatic glucose production, muscle glucose assimilation, appetite adjustment, and arterial inflammation through various adipocytokines, such as adiponectin, leptin, resistin, and tumor necrosis factor-alpha (TNF-alpha). A process of increased lipolysis occurs at the level of visceral fat. Through this process, the flow of free acids in the liver increases, insulin resistance also increases, and there is high production of abnormal lipid particles, mainly triglycerides [10,11].

The average systolic and diastolic blood pressure increases significantly with increasing BMI. Vascular remodeling, with a significant role in arterial hypertension, occurs due to neurohormonal processes caused by excess adiposity. There is stimulation of the renin–angiotensin system (RAS), leptin activity, and the sympathetic nervous system (SNS) due to events with a pro-inflammatory role [3]. Meanwhile, cardiovascular pathologies, especially heart failure, are associated with and aggravated by obesity, with this playing a major role in the various models of remodeling of the left ventricle. Through the important chronic inflammation process, obesity contributes to the development and diversity of heart failure [16].

Second, obesity increases insulin resistance and raises circulating insulin levels, which can lead to type 2 diabetes [17]. Furthermore, obesity promotes an imbalance between glucagon-like peptide-1 (GLP-1) and glucagon-like peptide-2 (GLP-2), impairing the incretin axis, which contributes to the appearance of insulin resistance; therefore, this whole process becomes involved in a vicious cycle [3].

In addition to all these cardiometabolic diseases, obesity through chronic inflammatory syndrome predisposes an individual to a series of overall dangerous pathophysiological changes. Insulin resistance stimulates endothelin-1 production, which further promotes increased atherogenesis and vasoconstrictor tone. Obesity is often associated with an altered lipid profile, which also contributes to the atherogenic process [3,18].

## 5. Correlations between Obesity and Metabolic Syndrome

For a long period of time, obesity has been diversely measured, with questionable research results due to the limitations of the studies regarding the indirect obesity methods of measurement.

An overview of the variables included in the present review is shown in Table 1.

Ibrahim Q et al. studied the association between selected variables (visceral adipose tissue (VAT), waist circumference (WC), and waist-to-hip ratio (WHR) and health risk in male and female subjects using a bioelectrical impedance analyzer. The results showed that 28.3% of the males and 9.4% of the females were at risk of obesity measured through VAT, 16.9% of the males and 6% of the females were at risk of obesity measured through WC, while 27.5% of the males and 6% of the females were at risk of obesity measured through WHR, with a significant relationship between all variables for the male and female subjects. The findings suggested that the order of VAT, WHR, and WC was a strong predictor of obesity, with males being more prone to health risks than the female participants [19].

The first cells recruited to injury sites, including inflammatory factors, neutrophils, and lymphocytes, components of innate and adaptive immunity, are used as prognostic factors in diverse inflammatory diseases, along with the neutrophil-to-lymphocyte ratio (NLR). The natural response of circulating leukocytes to pathophysiological processes following stress or chronic systemic inflammation is reflected by the increase in neutrophils and the decrease in the number of lymphocytes [20]. An elevated NLR value is also a “significant negative predictor in cancer or atherosclerosis”. Determined from the results of blood samples, the NLR is a practical and widely available biomarker in the establishment of outcomes in many medical conditions. Through a study conducted by Rodriguez-Rodriguez et al. on a population of 1747 non-institutionalized adults over 50 years of age, the importance of the NLR as a novel biomarker was considered. 

Differences between the anthropometric measurements in men vs. women are represented in Table 2.

Statistically significant differences between the male and female groups were found regarding BMI, while highly statistically significant values were discovered regarding WC, WHtR, WHR, and body fat percentage. In both groups divided by inflammatory status according to NLR values with a cut-off point of NRL ≥ 2.8 in males and NLR ≥ 2.4 in the female subjects, anthropometric measurements related to obesity definition were statistically tested [21].

No significant differences were found between the groups with high versus low inflammatory status according to gender, although the men, as well as the women, with a more severe inflammatory status presented a higher level of WC, WHtR, and WHR (anthropometric markers of central obesity) in comparison with those who had a reduced inflammatory process [25].

The level of systemic inflammation can be determined by hematologic markers as a largely available and inexpensive inflammatory biomarker tool [20]. It has been concluded that central obesity leads to increased levels of inflammation, presented as an elevated NLR, whereas diet quality (men following diets rich in protein and women following diets rich in vegetables and cereals seem to be significant protective factors against a high inflammatory state) is associated with a lower inflammatory state [22,26].

Using human and mouse models, Russo et al. investigated the role of cholesterol 25-hydroxylase (CH25H) in metabolic inflammation through transcriptomic analysis (RNA-Seq) of human AT biopsies, including visceral adipose tissue (VAT, greater omentum) and subcutaneous adipose tissue (SAT, abdominal wall), obtained from bariatric surgery patients. To investigate whether CH25H was increased in DM, they selected adipose tissue macrophages from 50 non-diabetic (NDM) and 50 diabetic (DM) obese women, diagnosed based on HbA1C and fasting glucose levels. The RNA sequencing results showed that human VAT and SAT from obese DM subjects are enriched in inflammatory pathways. CH25H mRNA is upregulated in VAT from obese diabetic subjects and enriched in human AT macrophages. Strongly significant statistical results were observed between the DM and non-DM groups regarding the differences between HbA1C and fasting glucose levels [27,28]. 

After demonstrating that CH25H is a high-fat diet (HFD)-induced gene, Russo et al. assigned a pro-inflammatory role to 25-HC, given its effect on the expression of pro-inflammatory cytokines, such as TNF-alpha, and highlighted that CH25H is required for HFD-induced insulin resistance and adipose tissue inflammation in mice in their studies [21,29].

In a study population of 98 patients divided into lean control, morbidly obese patient (OB), and morbidly obese with metabolic syndrome patient (OB + MetS) groups before (0) and after bariatric surgery at 1, 3, 6, and 12 months, Choromanska et al. found strongly significant higher values in weight, BMI, WC, and WHR in every tested stage compared to the control group. Moreover, weight and BMI were strongly significantly lower in the OB group compared to the OB +MetS group at 3, 6, and 12 months after BS. Furthermore, a decrease in weight and central obesity anthropometrical measurements could be observed in every stage after BS compared to the baseline [30].

Strongly significantly higher values in the OB + MetS group at 3, 6, and 12 months compared to the OB group were also found, while the OB + MetS group at 6 and 12 months after BS had strongly significantly lower values compared to the baseline regarding fasting glucose, fasting insulin, and HOMA-IR score assessment. Significant differences between the morbidly obese with metabolic syndrome (OB + MetS 0) patients before BS were observed in a decreased level of TC at 12 months after BS and a decreased level of LDL cholesterol at 1, 3, 6, and 12 months after BS, while HDL cholesterol levels increased [31].

Another study by Choromanska et al. compared 50 morbidly obese female patients (OB) (with BMI > 40 kg/m^2^) aged 28 to 56, who underwent weight-loss surgery, subclassified into two groups of morbidly obese patients with metabolic syndrome (MetS+) and morbidly obese patients without metabolic syndrome (MetS−), with a control group consisting of 50 lean, healthy female patients. The blood tests for examination were collected before BS (OB 0), as well as at 1, 3, 6, and 12 months (OB 1, 3, 6, and 12) after BS. Comparing the anthropometric measurements, it was no surprise that they found strongly statistically significant higher values at 6 and 12 months after BS regarding the indicators of central obesity (weight, BMI, and WHR) in the morbidly obese group compared to the control group and statistically significantly lower values compared to the baseline (OB 0). Regarding the laboratory analysis results, they found statistically significantly higher values in systolic blood pressure (SBP) and glucose in the morbidly obese patient group compared with the lean controls, with a respective decrease in high-density lipoprotein (HDL) levels, progressively in every evaluated time frame. In comparison, strongly statistically significantly higher values in total cholesterol, low-density lipoprotein (LDL), insulin, and homeostatic model assessment of insulin resistance (HOMA-IR) were observed by comparing the same groups. Progressive improvement can be seen in each and every evaluated laboratory analysis as the months progress following BS [31,32]. 

In a retrospective study, comparing 24 morbidly obese patients undergoing sleeve gastrectomy (SG) with 20 healthy patients, evaluated at baseline and 6 months after surgery, Catoi et al. evaluated insulin resistance and lipid profile markers. Through comparison, they found strongly significant differences, such as higher levels in the OB group vs. the healthy controls regarding mean weight, BMI, HOMA-IR assessment score, and TGs, while significant differences were observed between the same groups regarding TC [33].

In another study, Catoi et al. focused on the analysis of inflammatory status following weight-loss surgery and quantified insulin resistance and lipid profile markers in a “metabolically healthy morbidly obese” patient group (MHMO) compared to a “metabolically unhealthy morbidly obese” patient group (MUHMO), selected according to the international metabolic syndrome criteria. The study was conducted on 72 morbidly obese (MO) patients divided into an MHMO group of 16 (22.22%), an MUHMO group of 32 with MetS (44.44%), and the other 24 patients were considered as part of the MUHMO group as they fulfilled only one criterion of MetS (33.33%). Regarding biochemical analysis pre- and post-operatively, there were strongly significantly higher values of glucose, insulin, and HOMA-IR score assessment, as well as significantly higher values of triglycerides and HDL-C in the MUHMO with the MetS group compared to the MHMO group. However, there were no statistically significant differences in TC and LDL-C between the two groups [34,35].

Catoi et al.’s study compared the two groups in terms of markers of inflammation, insulin resistance, and factors predicting MetS and found that insulin and HOMA-IR score were significantly lower in the MHMO group than in the MUHMO group, whereas fasting insulin and HOMA-IR score were identified as possible predictors of MetS in the MHMO group [23,32].

A prospective study by Min et al., comprising 19 morbidly obese individuals, aimed to evaluate biochemical changes in obesity-related inflammatory status at 1 and 6 months, as well as 4 years, after BS. Seventeen out of the nineteen participants had type 2 diabetes mellitus, while the two other participants had impaired fasting glucose pre-operatively. Regarding anthropometric and clinical evaluation, a significant decrease in body weight and BMI were observed after 6 months, with maintenance observed at 4 years after weight-loss surgery. A significant decrease in blood pressure and an increase in HDL cholesterol levels were observed. Regarding insulin sensitivity, HbA1c showed a significant reduction, while there were no significant differences in the levels of insulin and HOMA-IR score assessment. However, the authors confirmed that 17.6% of the participants had achieved complete diabetes remission after 4 years [24,34].

## 6. Correlations between Obesity and Inflammatory Induced Comorbidities

### 6.1. Inflammation in Heart Failure

Chronic, low-grade inflammation is also a common feature of heart failure (HF). Inflammatory markers were highest among obese patients with the highest BMIs, which was the group with the shortest event-free survival. This is consistent with previous findings that show that the level of inflammation is related to the individual’s BMI. These data suggest that inflammation is a potential mechanism to explain why a greater BMI is associated with worse outcomes in those patients with HF. Saleh et al. investigated the hypothesis that higher levels of pro-inflammatory mediators, particularly TNF-alpha, are associated with greater HF severity and worse HF prognosis. Research was conducted on 415 patients divided into 4 groups based on their BMI and a median split of soluble tumor necrosis factor receptor1 (sTNFR1) levels as follows: (1) obese with high inflammation: a BMI above 30 kg/m^2^ and sTNFR1 above 1804 pg/mL; (2) obese with low inflammation: a BMI above 30 kg/m^2^ and sTNFR1 under 1804 pg/mL; (3) lean with high inflammation: a BMI under 30 kg/m^2^ and sTNFR1 above 1804 pg/mL; and (4) lean with low inflammation: a BMI under 30 kg/m^2^ and sTNFR1 under 1804 pg/mL. The patients in the high-inflammation groups were older and had higher comorbidity scores and NT-proBNP levels than those in the low-inflammation groups. The obese/high-inflammation group had a higher BMI and a greater number in NYHA class III/IV than all other groups. The patients in the high-inflammation groups had a greater number of events of all-cause hospitalization or death and a shorter median time to first event-free survival than those in the low-inflammation groups [35]. 

Obese patients with HF who have a higher level of inflammation are at a higher risk of being hospitalized or dying than other patients with HF. The levels of NT-proBNP were higher in the non-obese/higher inflammation group than in any of the other groups. Obese patients with heart failure are known to have lower NT-proBNP levels compared to non-obese patients. This suggests that inflammation is associated with higher NT-proBNP levels. The results showed that the obese patients with HF and a higher inflammatory response had shorter event-free survival compared with the non-obese patients with a lower inflammatory response [35,36].

### 6.2. Glutamine Metabolism

Glutamine metabolism seems to be linked to white adipose tissue (WAT) inflammation in obesity and associated metabolic complications. Petrus et al. reported glutamine to be an immune–metabolic regulator in WAT that links obesity and inflammation. In a study of 81 obese and non-obese female patients, they demonstrated that increased fat mass and fat cell size display significantly lower glutamine levels (*p* < 0.01). Studies performed in vitro by the same authors on differentiated adipocytes showed altered expression of glutamine-metabolizing genes in human adipose tissue in obesity, while high glutamine levels attenuated leukocytosis, glycolysis, and inflammation through the IL-6 and IL-1β pathways. Moreover, their data confirmed that glutamine exerts anti-inflammatory effects on macrophages and T cells, and this extends to include differentiated adipocytes as well as WAT in vivo. Glutamine metabolism is disturbed in the WAT of obese individuals and correlates with a harmful WAT phenotype. Reduced glutamine levels shift the balance from glutaminolysis to glycolysis. This leads to nuclear O-GlcNAcylation, which activates inflammation [37].

## 7. Correlations between Obesity and Pro-Inflammatory Status

Levels of pro-inflammatory mediators (IL-6 and TNF-alpha), the main factors responsible for inducing the production of acute-phase proteins (CRP), are associated with the prognosis and severity of cardiovascular disease [38]. A study conducted by K. Popko et al. confirmed that elevated levels of both cytokines induce persistent inflammation in obese individuals. However, the development of an obesity-related inflammatory state may be affected by other factors, such as gender [39]. 

Hypothesizing that inflammation, represented through increased values of IL-6 and CRP, correlated with leptin in patients following acute myocardial infarction, could be due to the involvement of leptin in the signaling cascade following myocardial ischemia, the study of Karthick et al. showed a relationship between serum leptin and IL-6 levels, demonstrating leptins’ involvement in inflammatory cytokine upregulation during heart ischemia [39,40].

Phillips et al. reported a positive benefit of weight loss through surgical procedures after a period of weight stabilization. Because of the cell diversity that produces the same cytokines, body weight loss as a result of bariatric surgery improves one specific set of cytokine-producing cells’ metabolic phenotype, causing a reduction in a specific cytokine from that tissue, but this may drive production in another cell type in the short term [40,41]. 

MicroRNAs (miRNAs) are gene-regulatory molecules involved in intercellular and inter-organ communication that have been shown to have important roles in many diseases, through endogenous processes that post-transcriptionally regulate gene expression. It was hypothesized that miRNA levels in adipose tissue, which plays a key role in obesity-related metabolic dysfunction, would change after gastric bypass surgery and that this would provide insights into their role in obesity-induced metabolic dysregulation. Recent studies have uncovered several miRNAs expressed in metabolic organs that could be used as possible therapeutic targets for obesity and its consequences, such as let-7, the potent regulator of glucose metabolism and peripheral insulin resistance, which showed significant alteration in obesity and metabolic disorders. MicroRNAs are emerging as new mediators in the regulation of adipocyte physiology and have been proven to play a role in obesity. Several studies have focused on miRNA expression profiles and functions in different metabolic tissues, which provides further evidence of the significant role of nutrition as an epigenetic factor in the regulation of lipid and glucose metabolism genes by modulating related key miRNAs; therefore, we suggest that miRNAs could be used as biomarkers for adiposity during diet-induced obesity [42].

Our narrative review attempted to add new insights to the present body of literature concerning the role of chronic inflammation in obesity and/or the metabolic syndrome pathogenic pathway. In doing so, we strove to include the most recent and trustworthy English-language papers published in recent years. Even so, our study has several limitations: firstly, there is heterogeneity among the included studies regarding the scientific quality of each study. Secondly, due to the large number of studies on the topic, the probability of selection bias must also be considered. 

## 8. Conclusions

This narrative review provides a comprehensive summary of the current understanding of the relationship between obesity, metabolic syndrome, and weight-loss effects. Weight-loss strategies such as physical exercise, dietary or pharmacological treatments, and surgical methods provide the possibility of relieving the burden of inflammation.

In line with previously published studies, it has been demonstrated that an improvement in BMI, glycemic control, and cardiovascular risk factors, with a decrease in acute-phase inflammatory proteins and pro-inflammatory markers, can be achieved by fast and substantial weight loss.

## Figures and Tables

**Table 1:** Overview of variable included in this review (published results in the literature).

Reference	Duration (y)	Study Type	Gender	Number of Patients	Study Groups (Average Age)	Objectives	Analyzed Markers	Indicator of MetSyn
Ibrahim et al. [19]	2017–2018 (2)	Cross-sectional analytical study	Men—240 Women—240	159	Men (19.17 ± 2.54) Women (18.96 ± 1.18)	Association between selected variables and health risk	BMI, WC, HC, VCA, AC, WHR, VAT	
Rodriguez-Rodriguez et al. [20]	2011 (1)	Observational and cross-sectional study	Men—771 Women—976	1747	Men (65.49 ± 10.45) Women (66.79 ± 10.76)	Association between selected variables and inflammatory state	BMI, WC, HC, WHtR, WHR,	Neutrophils-Lymphocytes(NLR), Inflammation status, Body Fat
Russo et al. [21]	2018 (1)	Cross-sectional study	Men—50 Women—50	100	50% DM (40.4 ± 11.8) 50% non-DM (46.6 ± 11.0)	Role of CH25H in metabolic inflammation	BMI, WC, HC	HbA1C and HOMA-IR, Cholesterol 25-Hydroxylase (CH25H); 25-Hydroxycholesterol (25-HC),
Choromansk et al. [22]	2012–2018 (7)	Cross-sectional study	Women—65	65	34—OB (39) 31—OB + MetS (49)	Insulin resistance and lipid profile markers	BMI, WC, HC, WHR,	LIPID PROFILE(HDL, TG, LDL, CHOLESTEROL), CRP, ALT, AST, WBC, RBC, HGB, PLT, GLUCOSE, INSULIN, HOMA-IR, Risk of T2DM and HTA
Catoi el al. [23]	2014–2016 (3) 2009–2015 (7)	Prospective study	Male—8 Women—16	24 72	(Control- 36.38 ± 6.3; MO- 42.15 ± 6.86) (MHMO-40.4 ± 9.0; MUHMO- 43.3 ± 10.03)	Insulin resistance and lipid profile markers	BMI, TC, HDL-C, TG, EBMIL,	HOMA-IR, hs-CRP, Chemerin, TNF-alpha,
Min et al. [24]	4 years follow-up	Prospective study	Male—6 Women—13	19	4 groups: Pre-operatively, 1 and 6 months, and 4 years Mean age (50.4 ± 6.2)	Biochemical changes in obesity-related inflammatory status at 1 and 6 months, as well as 4 years after BS	BMI, EWL, SBP, DBP, TC,	Adiponectin, leptin, CRP, Il-6, Il-10, HbA1C, lipid profile(LDL, HDL, TG), Fasting C-peptide, Insulin, HOMA-IR

Abbreviations: AST—Aspartate Transaminase; ALT—Alanine Transaminase; AC—Abdominal Circumference; BMI—Body Mass Index; CH25H—Cholesterol 25-Hydroxylase; CRP—C-reactive protein; DBP—Diastolic Blood Pressure; DM—Diabetes Mellitus; EBMIL—Excess Body Mass Index Loss; EWL—Electronic Waist List; HbA1C—Hemoglobin A1C; HGB—Hemoglobin; HC—Hip Circumference; HDL—High-Density Lipoprotein; HDL-C—High-Density Lipoprotein Cholesterol; HOMA-IR—Homeostatic Model Assessment for Insulin Resistance; HTA—Arterial Hypertension; hsCRP—high-sensitivity C-Reactive Protein; IL 1,6,10—Interleukin 1, 6 and 10; LDL—Low-Density Lipoprotein; MO—Morbid Obese; MeS—Metabolic Syndrome; MUHMO—Unhealthy Morbidly Obese Patients Group; NLR—Neutrophil-to-Lymphocyte Ratio; OB—Obesity; PLT—Platelet Count; RBC—Red Blood Cell; SBP—Systolic Blood Pressure; T2DM—Type 2 Diabetes; TC—Total Cholesterol; TG—Triglyceride; VAT—Visceral Adipose Tissue; WAT—White Adipose Tissue; VCA—Cross-sectional Visceral Compartment Area; WBC—White Blood Cell; WC—Waist Circumference; WHR—Waist-to-Hip Ratio; WHtR—Waist-to-Height Ratio.

**Table 2:** Biochemical characteristic at baseline of the analyzed groups of each mentioned journal.

Ibrahim et al. [19]	Men	Women				Both		
Age	19.17 ± 2.54	18.96 ± 1.18				19.07 ± 1.99		
Height	167.57 ± 8.38	157.22 ± 5.27				162.40 ± 8.71		
Weight	76.888 ± 27.89	56.52 ± 12.26				66.70 ± 23.81		
BMI	76.888 ± 27.89	22.86 ± 4.81				24.98 ± 7.32		
VCA	103.738 ± 72.26	55.95 ± 32.63				79.84 ± 60.89		
AC	93.89 ± 22.17	76.29 ± 8.97				85.09 ± 19.05		
WHR	93.89 ± 22.17	76.29 ± 8.97				0.87 ± 0.98		
Rodriguez-Rodriguez et al. [20]	Men (*n* = 771)	Women (*n* = 976)				*p* value		
BMI (kg/m^2^)	28.55 ± 4.07	28.20 ± 4.98				*p* < 0.05		
WC (cm)	100.67 ± 10.42	94.79 ± 12.75				*p* < 0.001		
WHtR	0.60 ± 0.06	0.61 ± 0.09				*p* < 0.01		
WHR	0.97 ± 0.07	0.91 ± 0.08				*p* < 0.001		
Body Fat (%)	30.63 ± 6.16	36.96 ± 7.61				*p* < 0.001		
Neutrophils (1000/mm^3^)	4.00 ± 1.33	3.75 ± 1.27				*p* < 0.001		
Lymphocytes (1000/mm^3^)	2.03 ± 0.70	2.08 ± 0.64				*p* < 0.01		
NLR	2.15 ± 0.96	1.93 ± 0.82				*p* <0.001		
Russo et al. [21]	Non-DM (*n* = 10)	DM (*n* = 10)				*p* value		
BMI (kg/m^2^)	48.31 ± 7.6	48.44 ± 6.3				0.946		
HbA1C (%)	5.61 ± 0.32	6.92 ± 1.21				0.0006		
Glucose (mg/dL)	88.2 ± 9.2	134.4 ± 49.2				0.0038		
Choromanska et al. [22]		Control	OB 0	OB 1	OB 3	OB 6	OB 12	*p* values
Weight (kg)		62 (60.32–63)	124	113	100.5	90	81.5	<0.0001
			(121.5–131.1)	(106.4–115.8)	(96.6–104.9)	(86.4–93.2)	(78–84.8)	
BMI (kg/m^2^)		23 (23–23)	46 (45–48)	41 (40–43)	37 (36–40)	34 (32–35)	30 (29–32)	<0.0001
WHR		0.72 (0.71–0.72)	0.97	0.98	0.97	0.96	0.92	<0.0001
			(0.96–0.99)	(0.96–0.99)	(0.94–0.99)	(0.93–0.97)	(0.91–0.94)	
SBP (mmHg)		120 (110–120)	130 (125–140)	130 (125–140)	130 (125–135)	128 (120–135)	125 (120–130)	<0.0001
DBP (mmHg)		80 (70–80)	85 (80–90)	85 (80–90)	80 (80–90)	80 (80–85)	80 (80–85)	<0.0001
TC (mmol/L)		175 (170–178)	198 (186–209)	185 (175–192)	177 (173–184)	184 (174–191)	175 (167–180)	<0.0001
LDL (mmol/L)		118 (116–120)	137 (128–148)	115 (110–120)	112 (104–119)	109 (99–118)	103 (99–113)	<0.0001
HDL (mmol/L)		60 (59–62)	46 (42–54)	45 (39–49)	47 (43–49)	50 (48–54)	55 (51–57)	<0.0001
TG (mmol/L)		134 (130–135)	135 (125–151)	126 (107–139)	115 (103–135)	116 (99–124)	98 (85–126)	<0.0001
Glucose (mg/dL)		76 (73–78)	101 (95–106)	98 (91–99)	93 (90–97)	93 (87–96)	87 (85–92)	<0.0001
Insulin (mUI/dL)		7.6 (7.4–7.8)	19 (17–22)	13 (9.8–15)	9 (7.3–11)	8.6 (7.5–9.2)	7.8 (6.9–8.5)	<0.0001
HOMA-IR		1.4 (1.3–1.5)	4.4 (4–5.4)	3 (2.4–3.5)	2 (1.7–2.4)	1.9 (1.7–2.2)	1.7 (1.5–1.9)	<0.0001
Catoi et al. [23]		Control group	Morbidly obese group			*p*-value		
Glucose (mg/dL)		90.75 ± 7.19	104.15 ± 21.06			0.093		
Insulin (mg/dL)		6.84 ± 3.64	19.28 ± 19.09			0.215		
HOMA-IR		1.43 (0.78–2.08)	3.31(2.66–5.05)			0.008		
Triglycerides (mg/dL)		80.50 (69.50–89)	122 (116–173)			0.004		
TC (mg/dL)		150 ± 22.82	187.35 ± 43.08			0.032		
HDL-chol (mg/dL)		40.18 ± 8.71	49.66 ± 15.81			0.135		
LDL-chol (mg/dL)		93.80 ± 19.34	112 ± 39.83			0.242		
Min et al. [24]		Baseline		1 month	6 months	4 years		*p* value
Weight (kg)		150 ± 37		132 ± 32	117 ± 29	116 ± 27		<0.001
BMI (kg/m^2^)		54 ± 14		48 ± 12	43 ± 11	43 ± 11		<0.001
SBP (mmHg)		137 ± 25		123 ± 15	131 ± 14	129 ± 20		0.021
DBP (mmHg)		81 ± 14		71 ± 10	76 ± 8	73 ± 13		0.001
TC (mmol/L)		4.3 ± 0.8		3.8 ± 1.1	4.2 ± 1.3	4.6 ± 1.3		0.178
LDL (mmol/L)		2.3 ± 0.6		2.1 ± 0.9	2.5 ± 1.2	2.4 ± 1.0		0.416
HDL (mmol/L)		1.2 ± 0.3		1.1 ± 0.3	1.2 ± 0.3	1.5 ± 0.6		0.002
TG (mmol/L)		1.7 ± 0.9		1.5 ± 0.5	1.4 ± 0.5	1.3 ± 0.5		0.151
Glucose (mg/dL)		7.3 (5.9–9.2)		5.7 (4.8–6.8)	5.4 (4.5–6.9)	6.4 (5.5–9.0)		0.378
Insulin (mU/L)		27.7 (19.6–38.6)		12.6 (7.6–25.6)	10.3 (6.0–20.7)	16.9 (6.0–34.4)		0.135
HbA1c (mg/dL)		58 ± 18		47 ± 12	45 ± 15	46 ± 15		0.049
HOMA-IR assessment score		0.4 ± 0.2		0.2 ± 0.3	0.1 ± 0.4	0.2 ± 0.4		0.098

## References

[1] WHO Regional Office for Europe. https://www.who.int/europe/publications/i/item/9789289057738.

[2] Vasheghani M., Hessami Z., Rekabi M., Abedini A., Qanavati A. (2022). Evaluating Possible Mechanisms Linking Obesity to COVID-19: A Narrative Review. Obes. Surg..

[3] Triposkiadis F., Xanthopoulos A., Starling R.C., Iliodromitis E. (2022). Obesity, inflammation, and heart failure: Links and misconceptions. Heart Fail. Rev..

[4] Wang M., Liu J., Zhang Z., Zhang H., Wang N., Chen X., Han X., Lu Q., Chi S. (2022). Effects of Dietary Intervention on Inflammatory Markers in Metabolic Syndrome: A Systematic Review and Meta-Analysis. Front. Nutr..

[5] Haddaway N.R., Page M.J., Pritchard C.C., McGuinness L.A. (2022). PRISMA2020: An R package and Shiny app for producing PRISMA 2020-compliant flow diagrams, with interactivity for optimized digital transparency and Open Synthesis. Campbell Syst. Rev..

[6] Lam D.W., LeRoith D. (2019). Metabolic Syndrome. Comprehensive Free Online Endocrinology Book.

[7] Nilsson P.M., Tuomilehto J., Ryden L. (2019). The metabolic syndrome—What is it and how should it be managed?. Eur. J. Prev. Cardiol..

[8] Barati E., Ghazizadeh H., Sadabadi F., Kazemi E., Ferns G.A., Avan A., Ghayour-Mobarhan M. (2019). Association of the IL6 Gene Polymorphism with Component Features of Metabolic Syndrome in Obese Subjects. Biochem. Genet..

[9] Hamdy O., Uwaifo G.I., Oral E.A. (2021). Obesity. Medscape.

[10] Gârgavu S.R., Clenciu D., Roșu M.M., Țenea Cojan T.Ș., Costache A., Vladu I.M., Moța M. (2018). Visceral adiposity index (vai)—A potential marker of cardiometabolic risk. Arch. Balk. Med. Union.

[11] Jameson J.L. (2013). Harrison’s Endocrinology.

[12] Eley V.A., Thuzar M., Navarro S., Dodd B.R., Zundert A.A.V. (2021). Obesity, metabolic syndrome, and inflammation: An update for anaesthetists caring for patients with obesity. Anaesth. Crit. Care Pain Med..

[13] Kaisanlahti A., Glumoff T. (2019). Browning of white fat: Agents and implications for beige adipose tissue to type 2 diabetes. J. Physiol. Biochem..

[14] Lidell M.E., Betz M.J., Enerbäck S. (2014). Brown adipose tissue and its therapeutic potential. J. Intern. Med..

[15] Cheng L., Wang J., Dai H., Duan Y., An Y., Shi L., Lv Y., Li H., Wang C., Ma Q. (2021). Brown and beige adipose tissue: A novel therapeutic strategy for obesity and type 2 diabetes mellitus, National Library of Medicine. Adipocyte.

[16] Mach F., Baigent C., Catapano A.L., Koskinas K.C., Casula M., Badimon L., Chapman M.J., De Backer G.G., Delgado V., Ference B.A. (2020). 2019 ESC/EAS Guidelines for the management of dyslipidaemias: Lipid modification to reduce cardiovascular risk. Eur. Heart J..

[17] Gallagher E.J., LeRoith D., Karnieli E. (2011). The Metabolic Syndrome—From Insulin Resistance to Obesity and Diabetes. Med. Clin. N. Am..

[18] Dieny F.F., Tsani A.F.A., Suryawati S. (2022). Visceral Adiposity Index and Lipid Accumulation Product Related to Insulin Resistance and Metabolic Syndrome in Obese College Students. Open Access Maced. J. Med. Sci..

[19] Ibrahim Q., Ahsan M. (2019). Measurement of Visceral Fat, Abdominal Circumference and Waist-hip Ratio to Predict Health Risk in Males and Females. Pak. J. Biol. Sci..

[20] Rodríguez-Rodríguez E., López-Sobaler A.M., Ortega R.M., Delgado-Losada M.L., López-Parra A.M., Aparicio A. (2020). Association between Neutrophil-to-Lymphocyte Ratio with Abdominal Obesity and Healthy Eating Index in a Representative Older Spanish Population. Nutrients.

[21] Russo L., Muir L., Geletka L., Delproposto J., Baker N., Flesher C., O’Rourke R., Lumeng C.N. (2020). Cholesterol 25-hydroxylase (CH25H) as a promoter of adipose tissue inflammation in obesity and diabetes. Mol. Metab..

[22] Choromańska B., Myśliwiec P., Łuba M., Wojskowicz P., Myśliwiec H., Choromańska K., Dadan J., Żendzian-Piotrowska M., Zalewska A., Maciejczyk M. (2020). Bariatric Surgery Normalizes Protein Glycoxidation and Nitrosative Stress in Morbidly Obese Patients. Antioxidants.

[23] Cӑtoi A.F., Pârvu A.E., Mironiuc A., Chiorescu Ş., Crӑciun A., Pop I.D., Cӑtoi C. (2018). Chemerin, Inflammatory, and Nitrooxidative Stress Marker Changes Six Months after Sleeve Gastrectomy. Oxidative Med. Cell. Longev..

[24] Min T., Prior S.L., Dunseath G., Churm R., Barry J.D., Stephens J.W. (2020). Temporal Effects of Bariatric Surgery on Adipokines, Inflammation and Oxidative Stress in Subjects with Impaired Glucose Homeostasis at 4 Years of Follow-up. Obes. Surg..

[25] Aratani Y. (2018). Myeloperoxidase: Its role for host defense, inflammation, and neutrophil function. Arch. Biochem. Biophys..

[26] Adiels M., Olofsson S.-O., Taskinen M.-R., Bore J. (2008). Overproduction of Very Low–Density Lipoproteins Is the Hallmark of the Dyslipidemia in the Metabolic Syndrome. Arterioscler. Thromb. Vasc. Biol..

[27] Yang Y., Shields G.S., Guo C., Liu Y. (2018). Executive function performance in obesity and overweight individuals: A meta-analysis and review. Neurosci. Biobehav. Rev..

[28] Russo L., Lumeng C.N. (2018). Properties and functions of adipose tissue macrophages in obesity. Immunology.

[29] Gold E.S., Diercks A.H., Podolsky I., Podyminogin R.L., Askovich P.S., Treuting P.M., Aderem A. (2014). 25-Hydroxycholesterol acts as an amplifier of inflammatory signaling. Proc. Natl. Acad. Sci. USA.

[30] Choromańska B., Myśliwiec P., Łuba M., Wojskowicz P., Dadan J., Myśliwiec H., Choromańska K., Zalewska A., Maciejczyk M. (2020). A Longitudinal Study of the Antioxidant Barrier and Oxidative Stress in Morbidly Obese Patients after Bariatric Surgery. Does the Metabolic Syndrome Affect the Redox Homeostasis of Obese People?. J. Clin. Med..

[31] Cho K.W., Zamarron B.F., Muir L.A., Singer K., Porsche C.E., DelProposto J.B., Geletka L., Meyer K.A., O’Rourke R.W., Lumeng C.N. (2016). Adipose Tissue Dendritic Cells Are Independent Contributors to Obesity-Induced Inflammation and Insulin Resistance. J. Immunol..

[32] Cӑtoi A.F., Pârvu A.E., Andreicuț A.D., Mironiuc A., Crӑciun A., Cӑtoi C., Pop I.D. (2018). Metabolically Healthy versus Unhealthy Morbidly Obese: Chronic Inflammation, Nitro-Oxidative Stress, and Insulin Resistance. Nutrients.

[33] Freitas W.R., Oliveira L.V.F., Perez E.A., Ilias E.J., Lottenberg C.P., Silva A.S., Urbano J.J., Oliveira M.C., Vieira R.P., Ribeiro-Alves M. (2018). Systemic Inflammation in Severe Obese Patients Undergoing Surgery for Obesity and Weight-Related Diseases. Obes. Surg..

[34] Stephens J.W., Min T., Dunseath G., Churm R., Barry J.D., Prior S.L. (2019). Temporal effects of laparoscopic sleeve gastrectomy on adipokines, inflammation, and oxidative stress in patients with impaired glucose homeostasis. Surg. Obes. Relat. Dis..

[35] Saleh Z.T., Lennie T.A., Darawad M., Alduraidi H., Elshatarat R.A., Almansour I.M., Moser D.K. (2020). The health outcomes of inflammation and obesity in patients with heart failure. Heart Lung.

[36] Farkhondeh T., Llorens S., Pourbagher-Shahri A.M., Ashrafizadeh M., Talebi M., Shakibaei M., Samarghandian S. (2020). An Overview of the Role of Adipokines in Cardiometabolic Diseases. Molecules.

[37] Petrus P., Lecoutre S., Dollet L., Wiel C., Sulen A., Gao H., Tavira B., Laurencikiene J., Rooyackers O., Checa A. (2020). Glutamine Links Obesity to Inflammation in Human White Adipose Tissue. Cell Metab..

[38] Rajendran K., Devarajan N., Ganesan M., Ragunathan M. (2012). Obesity, Inflammation and Acute Myocardial Infarction—Expression of leptin, IL-6 and high sensitivity-CRP in Chennai based population. Thromb. J..

[39] Popko K., Gorska E., Stelmaszczyk-Emmel A., Plywaczewski R., Stoklosa A., Gorecka D., Pyrzak B., Demkow U. (2010). Proinflammatory cytokines IL-6 and TNF-α and the development of inflammation in obese subjects. Eur. J. Med. Res..

[40] Beresescu G., Sala D.T., Ion R.M., Tegla E., Balmos A., Cosarca A., Ormenisan A. (2019). Relationship Between Obesity and Periodontal Disease after Minimally Invasive Sleeve Gastrostomy. Rev. Chim..

[41] Phillips C.L., Grayson B.E. (2020). The immune remodel: Weight loss-mediated inflammatory changes to obesity. Exp. Biol. Med..

[42] Youssef E.M., Elfiky A.M., Abu-Shahba N. (2020). and Elhefnawi, M.M. Expression profiling and analysis of some miRNAs in subcutaneous white adipose tissue during development of obesity. Genes Nutr..

